# Aphasia Depression and Psychological Therapy (ADaPT): Perspectives of People with Post-Stroke Aphasia on Participating in a Modified Cognitive Behavioral Therapy

**DOI:** 10.3390/healthcare12070771

**Published:** 2024-04-02

**Authors:** Caroline Baker, Sonia Thomas, Priscilla Tjokrowijoto, Brooke Ryan, Ian Kneebone, Renerus Stolwyk

**Affiliations:** 1 Speech Pathology Department, Monash Health, Melbourne, VIC 3192, Australia; 2 Centre of Research Excellence in Aphasia Recovery and Rehabilitation, Melbourne, VIC 3086, Australia; priscilla.tjokrowijoto@mh.org.au (P.T.); brooke.ryan@curtin.edu.au (B.R.); ian.kneebone@uts.edu.au (I.K.); rene.stolwyk@monash.edu (R.S.); 3 Thinking Matters, Melbourne, VIC 3184, Australia; thinkingmattersonline@gmail.com; 4 Turner Institute for Brain and Mental Health, School of Psychological Sciences, Monash University, Melbourne, VIC 3800, Australia; 5 Stroke and Telehealth Research, Monash-Epworth Rehabilitation Research Centre, Melbourne, VIC 3121, Australia; 6 Speech Pathology, Curtin School of Allied Health, Curtin University, Perth, WA 6845, Australia; 7 Graduate School of Health, University of Technology Sydney, Sydney, NSW 2007, Australia

**Keywords:** stroke, aphasia, modified cognitive behavioral therapy, psychological therapy, stroke rehabilitation

## Abstract

Aphasia, a communication disability commonly caused by stroke, can profoundly affect a person’s mood and identity. We explored the experiences of stroke survivors with aphasia and depression who received a modified cognitive behavioral therapy (CBT)-based psychological intervention. The therapy is manualized with a flexible treatment protocol, including 10 individually based therapy sessions (+2 booster sessions) either via telehealth or in person. Six participants with chronic aphasia (60% of the total sample) participated in in-depth interviews that were analyzed using reflexive thematic analysis. Two core themes were derived from the data: the first theme, helpful elements of therapy—doing enjoyable activities, new ways of thinking, problem solving, working with the experienced therapist, and using telehealth; and the second theme, making progress—mood, communication, acceptance of the ‘new me’, and improving relationships. All participants found the therapy to be helpful in managing mood problems with various elements being beneficial depending on the individual, highlighting the importance of tailoring the intervention. Therefore, delivering modified CBT to individuals with aphasia is likely to be acceptable both in person and through telehealth. Further evaluation of the intervention and its impact on mood would be beneficial.

## 1. Introduction

Acquired communication disabilities after stroke are common and can include aphasia (difficulties with verbal and written expressions, understanding, and reading); apraxia of speech/dysarthria (motor speech problems); and/or cognitive communication impairments (difficulties with conversational discourse and social skills) [1,2]. Approximately, a third of people who have a stroke experience aphasia [3]. Aphasia after stroke can have negative impacts on a person’s identity and relationships, which can lead to fewer friendships and less participation in social activities [4,5]. Depression and anxiety are common mood concerns after post-stroke aphasia [6,7], reported at higher rates compared to those without aphasia [6,7,8,9]. Despite some research efforts, there is still a need for psychotherapeutic interventions that are both acceptable and effective for the mental health and wellbeing of individuals with aphasia [10,11].

Cognitive behavioral therapy (CBT) is a psychotherapy treatment that helps a person to manage their emotions, optimize their everyday activities and functioning while maintaining realistic, yet optimistic thinking [12]. One primary aim of CBT is reducing symptoms of depression, such as apathy, hopelessness, and low mood across a range of clinical populations [12]. CBT can effectively treat depression in the general, neurotypical population with comparable effects to pharmacotherapy treatment [13]. Furthermore, it has emerging support for positive effects in reducing overall symptoms and maintaining improvement in those with stroke [14,15]. However, there is currently limited evidence for the use of a CBT intervention adapted to support the specific communication and psychological needs of stroke survivors with aphasia. Despite the known high prevalence rates of mood problems in people with aphasia, stroke audits and research evidence consistently report a lack of mood assessments and appropriate psychological care and follow-up treatment with mental health specialists [16,17]. The access and provision of psychological treatments after stroke is a high priority in the research and clinical care [17]. To address this significant gap in clinical care, Aphasia Depression and Psychological Therapy (ADaPT) was developed to test feasibility and preliminary efficacy for the treatment of depression after post-stroke aphasia using a single-case-design evaluation study [18]. ADaPT is a protocolized, tailored treatment program delivered by a clinical neuropsychologist. It focuses on approaches to support communication skills and access (e.g., supported conversations and photo diaries); behavioral skills (e.g., scheduling enjoyable activities and relaxation techniques); and cognitive skills (e.g., modifying negative thoughts and problem solving) [16]. The establishment of acceptability within ADaPT is essential.

The AdaPT treatment outcome study found that the intervention was largely feasible and could be implemented with most of the participants (n = 10) via telehealth with varying degrees of aphasia severity and time post-stroke [18]. The primary outcome measure was self-reported depression. Three of the participants reported an improvement in mood during the intervention phase, which was sustained for two of these participants during follow-up sessions. An additional four participants demonstrated a delayed treatment response during the follow-up period. Three participants did not appear to benefit during the study period, one of which did not complete all of the intervention sessions [18].

This current study considered the retrospective (experienced) acceptability of ADaPT by examining patient perspectives as the participants of the intervention [19] as an additional qualitative analysis approach to the quantitative results report of the single-case series evaluation [18]. When patients find a therapy acceptable and tailored to their needs, they are more likely to engage fully, attend all sessions, and apply the strategies they learn, which is vital for achieving positive outcomes [19]. Therefore, this qualitative study followed the completion of delivery of CBT within the ADaPT single-case series study [18]. Qualitative methodology was an appropriate approach to explore the experiences of people with aphasia who participated in this modified CBT as part of an evaluation of its feasibility. It can provide an in-depth understanding of the person’s experience (e.g., self-perceived helpful or unhelpful aspects of treatment), which can complement the findings gained from quantitative measures.

Specifically, the aims of the study were to: (1) explore the experiences of people with aphasia when participating in modified CBT; (2) identify their needs and preferences; and (3) provide recommendations to improve the therapy.

## 2. Materials and Methods

### 2.1. Design

Reflexive thematic analysis was chosen for the methods and data analysis as an appropriate approach to this qualitative study due to the purpose of exploring participants’ experiences and perspectives of therapy. This is a common approach used in health science research as it values the researcher’s subjective experience as the primary way to discern knowledge from the data. The purpose is to derive meaning and sense of the data from using the researcher’s experiences and values, rather than aiming to search for objectivity and remove bias [20,21]. The approach has been used in previous research on aphasiology, for example, in exploring prognostication in post-stroke aphasia, and also in treatment studies, for example, in exploring experiences of interventions in healthcare [22,23]. Individual interviews were chosen to enable participants to have their experiences conveyed in a supportive communication environment with the researcher (CB) [24]. The research followed the Consolidated criteria for Reporting Qualitative research (COREQ) [25] (see Supplementary File Table S1).

### 2.2. Participant Recruitment and Eligibility

All 10 participants who completed all therapy sessions of ADaPT were considered for inclusion and invited to take part in an in-depth interview within 6 weeks of completion of the modified CBT intervention [18]. Six were interviewed; three participants declined (two due to a decline in their medical condition) and one was considered to decline as they failed to respond to 3 invitations. To be eligible to participate in the ADaPT study [18], participants had to be adults aged 18 years or older; had a diagnosis of ischemic/hemorrhagic stroke confirmed by a health practitioner; a clinical diagnosis of aphasia (Western Aphasia Battery-Revised (WAB-R) < 93.7) [26]; a self-reported low mood (≥2 on the Depression Intensity Scale Circles (DISCs)) [27]; no previous history or concurrent major neurological/psychiatric diagnosis; capacity to consent (with supported communication); capacity and availability to engage; and not concurrently receiving any other psychological interventions and on stable dose of mood medications, if any. Participants were identified through the community, stroke, and aphasia organizations (e.g., social media and mail-out flyers via the Stroke Foundation and professional mailing lists) [18]. Those consenting to an interview were allocated a unique code to protect anonymity, identified throughout this manuscript using their code (P1–P6). Four participants were male and two were female, aged 58 to 71 years. All except one participant lived with a family member. Participants 1, 2, and 3 were interviewed with their spouse/carer present who provided communication support during the interview (Table 1). The mean duration of the interviews was 56 min (range: 45–75 min).

### 2.3. ADaPT Intervention Sessions

A full description of the intervention is provided [18]. In summary, ADaPT was tailored to the goals and needs of each participant using approaches of communication and cognitive support, behavioral activation, identity renegotiation, and cognitive therapy (challenging and modifying unhelpful thinking patterns/thoughts) [18]. The therapy sessions were conducted via a mix of in-person and telehealth sessions subject to COVID-19 pandemic restrictions and client preference.

### 2.4. Data Collection

The perspectives of participants with aphasia were gathered through semi-structured interviews. The topic guide was developed by the first author (CB) with revisions and suggestions offered by all co-authors (see Supplemental File Table S2 [28,29]). The topic guide included open-ended questions and prompts to support a semi-structured interview about the person’s experiences, needs, preferences, and recommendations regarding the ADaPT intervention. How they experienced study procedures was also a topic of the interview, but not reported in this current study. To facilitate the interviews, total communication strategies were used (e.g., using short and simple phrases, using written key words/pictures as needed, verifying and confirming the participant’s message, asking for clarification, repeating questions as needed, and engaging the support person as a communication partner) [30]. All interviews were conducted via Zoom at the participant’s home, except for one person who was interviewed in person in a clinic setting. All participants consented to video recording and the interviews were transcribed verbatim. The interviewer had previously met participants for their baseline WAB-R aphasia tests prior to commencing CBT. Otherwise, the interviewer remained independent of the participants’ involvement in the ADaPT study [18].

### 2.5. Data Analysis

The interview data were analyzed inductively using reflexive thematic analysis [20,21] with coding and derivation of themes from across the dataset (see Supplemental File Table S3 for an example of the reflexive thematic data analysis process). The analysis followed the following steps: familiarization of video/audio recordings and transcripts with repeated readings and note-taking to generate initial reflections (CB); open coding of all transcripts (CB); peer review of transcripts and codes of a third of the transcripts (ST); peer debriefing of codes (ST and CB); generation of themes within each transcript (CB); derivation of themes across transcripts (CB); peer debriefing of themes derived across the sample with all co-authors; refining, defining, and naming themes (CB); derivation of final core themes and subthemes (all authors); and producing the report (all authors) with exemplar quotes from participants with aphasia. All quotes were transcribed verbatim from the participant with aphasia, which may or may not have included language errors. The study complied with the research rigor described in Lyons and McAllister [31] (details in Supplementary File Table S1).

### 2.6. Ethical Statement

The Monash University Human Research Ethics Committee approved this study (HREC ID 7888). All participants provided verbal and/or written informed consent. Information and consent processes were provided in a communicatively accessible manner by the researcher (PT), consistent with stroke research inclusion recommendations for people with aphasia [32].

## 3. Results

The qualitative data show a complex and varied experience of participation in the ADaPT intervention, where, overall, the therapy was valued by participants and perceived as a helpful part of their aphasia and stroke rehabilitation and recovery. All participants reported that the therapy helped to manage mood problems in different ways (e.g., through doing enjoyable activities and/or modifying unhelpful thoughts). All participants perceived the benefits of managing their mood and other aspects of life (e.g., relationships).

Two core themes were derived from the qualitative data with perceptions around: (1) helpful elements of therapy and (2) making progress.

### 3.1. Core Theme 1: Helpful Elements of Therapy

Participants perceived the therapy sessions as personalized and adapted for their specific goals and needs at the time, rather than tasks set by the therapist to perform, regardless of their current mood and wellbeing needs. A participant particularly valued the ‘flexible’ ‘very person-centered’ approach to the ADaPT therapy. This approach led to personalized therapy goals and activities, such as writing to their grandchildren living at a distance from the participant:

‘She [therapist] was very person-centred…she sent me the picture [of participant’s holiday location] and so I was talking about the idea to connect to my grandchildren, and I’d thought of writing them a letter every two weeks. And so I’ve started doing that…’ (P5, female).

In terms of what the participants found particularly helpful, five subthemes were derived from the data: (a) doing enjoyable activities; (b) new ways of thinking; (c) problem solving; (d) working with the experienced therapist; and (e) using telehealth.

#### 3.1.1. Doing Enjoyable Activities

The majority of participants described how therapy helped them to consider and identify enjoyable activities, as a participant described it as:

‘the things that gave me joy’ (P5, female).

Sessions provided an opportunity to plan and schedule doing these activities, share how things went in the following session, and how this impacted on their mood. For example, a participant described how her valued activities were:

‘simple things…like nice smells [putting on the oil vaporizer]…heat and warmth…listening to music and dancing.’ (P5, female)

She described that the therapist helped her to focus on how far she had come and what she was able to do rather than unable to do. Another participant described going outdoors:

‘to see the birds, as well as the dogs.’ (P6, male)

He looked forward to the opportunity most days:

‘I’ll come across a couple of magpies, a mother and a baby…I get a bit of bread…I’ll talk to her and feed her…they take food out of my hand. So things like that are really good. That makes me happy.’ (P6, male)

When the person themselves or the therapist noted success in participating in such activities, it boosted their confidence and positive emotions. In contrast, for the same participant (P6, male), if activities were complex (e.g., boat improvements), there were mixed emotions due to only the partial completion of the activity:

‘I’ve got new carpet in the boat…I’m happy with that…[now] it’s just a matter of putting the seats back in and I just can’t be bothered.’ (P6, male)

Participants reported that the therapist helped them to manage physical and communication difficulties in order to participate in enjoyable activities. For example, knowledge and skill building with the therapist to prepare for planned activities for the week (P2, male discussing with his therapist going to a café with his spouse and a small group of friends). The therapist also facilitated connecting participants with other health professionals in the community, such as an occupational therapist and physiotherapist (P6, male). Some participants were restricted in their ability to participate in activities due to COVID-19. A participant reported not being able to do the things he would have liked to, which resulted in him feeling ‘a little bit worse’ in mood during this time:

‘…I was unable to get out…less freedom…It was bad. I feel myself lock in jail.’ (P3, male)

#### 3.1.2. New Ways of Thinking

Participants valued the explicit information provided by the therapist about negative thinking and how this way of thinking can be changed. All participants perceived value in the component of therapy that involved challenging and modifying their negative thoughts or unrealistic expectations of recovery. An example of how participants’ valued this aspect of ADaPT was shown in the following interaction with a participant with severe aphasia:

Interviewer: did anything surprise you in the therapy? Surprise.

Spouse: which one? [From choice on worksheets]. Oh what’s the evidence? [What is the evidence] I have to support my thoughts?

P6, male: [pointing to ‘What’s the evidence?’ worksheet]. ‘Yeah’.

Interviewer: that surprised you to think about that?

P6, male: ‘Yeah. Thank God, yeah, yeah.’

The participant’s spouse strongly endorsed the combined techniques of doing enjoyable activities and working through challenging-thoughts worksheets. She reported that the CBT was well-organized and well-explained; she was enabled to support the completion of home-practice tasks. She observed an improved mood, engagement, and confidence in her husband, particularly with him being more involved with family and friends in social situations. In contrast, two other participants found the ‘What is the evidence?’ concept and worksheet the least helpful part of their therapy. They were introduced to the concept, but preferred to focus on identifying and performing enjoyable activities that were supported by the therapist.

Another participant reported how the therapist had encouraged a different way of thinking, to achieve a more positive outlook:

‘[Therapist] asked me to help whichever I think, I am thinking about the good side, about positive side…rather than, I just thinking about the worst.’ (P3, male)

Modifying negative thoughts was considered the most helpful aspect of therapy for this participant. He reported perceived benefits in his life overall since completing ADaPT, indicating a positive impact (on a scale of 1–10, scoring 9) and with sustained improvements in mood and wellbeing:

‘I feel less worried…I feel more better.’ (P3, male)

Participants reported feeling enabled to incorporate this strategy into everyday routines or when needed. For example, a participant reported on their progress:

‘So, my cup was always half empty, always. I was never in a good mood…I’m still like that but I can understand things a bit better…[the therapist] helped me look at things differently, much differently’ (P6, male).

This participant was able to put strategies into place and noted improvements in working through problems and getting along with others as a:

‘big positive impact [after intervention] because I didn’t know how much I could change my thought for a better way, sees towards other people...’ (P6, male).

#### 3.1.3. Problem Solving

Participants reported facing different problems associated with a range of changes in functioning after stroke (e.g., physical, communication, and cognitive changes). They reported that the therapist helped them navigate these problems through counseling skills (e.g., active listening) and in practical ways, such as providing information resources, linking participants to community supports, and supporting their skills and confidence to use their problem-solving action plan (e.g., discussing the scenario in advance, such as a difficult conversation with their spouse). They often felt different and more vulnerable to others, particularly due to the ‘invisibility’ of the communication difficulties and the lack of awareness of others about stroke and communication difficulties. This was evident through descriptions provided by a participant preparing to return to driving:

‘At the time, with those people [Road Traffic Authority workers] were not very helpful and they did not understand aphasia very much at all.’ (P4, female)

This participant also experienced anxiety in what was required to return to driving her car after having a stroke. She perceived that the therapist enabled her to ‘get a positive thing’ from her interactions during this testing process as she felt supported with various strategies (e.g., break large task into small actions and relaxation techniques). These strategies were useful during and following the ADaPT intervention:

‘You know when you’re calm and she had different ways that I could go to do that, little things that I could do that…they helped me immediately with that and even now.’ (P4, female)

The feeling that the therapist understood participants’ problems and feelings was perceived to make a difference. Participants felt supported to take steps to work through current problems they faced rather than feeling alone.

Within ADaPT, some participants reported that home practice was difficult to complete. However, they felt supported by the therapist to complete tasks with support either from a family member or from therapy sessions with the therapist. For example, two participants were assisted by a close other (spouse/formal carer) to work through the tasks. Due to communication difficulties (talking, reading, and writing), a participant felt home practice was the least useful part of the intervention and rather completed parts of this within sessions with the therapist.

All participants perceived that the time commitment to CBT was significant but worthwhile, and that they would recommend the CBT treatment to others with aphasia. Some participants felt ‘busy’ with and challenged by other commitments (e.g., medical appointments and holidays), but were able to discuss and agree upon alternative therapy scheduling with the therapist. Strategies to overcome scheduling problems included offering telehealth from locations other than home (e.g., local library room), appointment reminders on the fridge, or via text message.

Some improvements to therapy were suggested by participants, which included: providing a clear understanding of the purpose of CBT to manage any potential expectations of language-based psychotherapy; simplifying some of the handouts around concepts of grief and loss/acceptance of changes in functioning post-stroke; and minimizing disruptions in the use of technology for telehealth.

#### 3.1.4. Working with the Experienced Therapist

The working relationship with the therapist was perceived to be an important element in the experience of participants. All the participants reported a highly positive experience working with the therapist, including over video conference, and noted her ability to understand aphasia and support conversations and emotions by allowing extra time and being open and approachable:

‘I think the relationship of a client to the therapist is an important factor…she listened. She took on board everything I said.’ (P5, female)

This participant also described the therapist as ‘flexible’. A participant noted that the therapist was the most useful part of the therapy:

‘I think it’s excellent [working with the therapist]…I have been really happy and very, really happy with [therapist]…she has helping me a lot with everything…she knew how to understand with depression and anxiety mainly…I think she understand as a psychologist, as a person who can really relate to what is happening there.’ (P4, female)

Another participant with a background in counseling prior to the stroke valued the ethics of CBT and thought it would be helpful prior to commencing the study.

Most participants appreciated the way that the therapist involved their support person in therapy, also noting her ability to explain concepts, such as modifying thoughts and using the worksheets, and ‘trouble shoot’ occasional technical breakdowns as needed with them.

Participants reported on the therapist’s ability to help them manage stress and anxiety through the use of a range of relaxation strategies. For example, they valued using technology, such as smartphones or iPads, to listen to audio recordings of relaxation exercises by the therapist. A participant reported:

‘There’ve been, for example for anxiety, there were different things that she’s [the therapist] done at home as well as recording to calm down…I’ll use those things and even get some apps as well…she’s [the therapist] given me a, how can I say? It’s a box…there of a whole lot of things that she’s helping me…’ (P4, female).

#### 3.1.5. Using Telehealth

All participants found it acceptable to use telehealth for participation in ADaPT, with most reporting progress in their familiarity and learning of how to use it across the course of the therapy. The Zoom platform was used by all participants, with only occasional difficulties with elements of the technology. A participant living interstate from the therapist valued being able to participate in the study via Zoom. He was supported by his spouse to use Zoom and participate in ADaPT due to severe aphasia:

Interviewer: Let’s talk about Zoom. What was it like to use Zoom? [offering rating scale].

P1, male: ‘Okay’ [pointing to the positive end of the rating scale].

The main reason described by participants that contributed to a positive view of telehealth was the convenience of not needing to travel and still being able to access the service despite living remote to the therapist. There were technical difficulties experienced by three participants, which led to some frustration and anxiety (e.g., difficulty working out how to access the Zoom link (P1, male) and the screen ‘freezing’ due to internet disconnection (P4, female). Despite this, most participants described being able to learn and grow in confidence in using Zoom. Three participants had a support person assist with the setup of Zoom (e.g., adjusting volume and accessing the meeting via the link). The therapist, using features such as screen sharing to support communication, was described as useful. A participant used an iPad and reported that a larger screen, such as a computer, would have been more helpful for seeing the therapist and the therapy worksheets (P3, male). All participants rated their interactions and working relationships with the therapist over Zoom as a positive experience.

### 3.2. Core Theme 2: Making Progress

Participants described the impact of their therapy as feeling ‘positive’, ‘more better’, ‘less worried’, ‘different to what I am or what I was’, and ‘better from before to now’. They also felt better equipped with strategies to manage not only mood problems, but other life challenges associated with communication difficulties (e.g., avoiding social gatherings), ‘to have the tools to try and do that [think positively]’. Subthemes included perceptions around (a) mood; (b) communication; (c) an acceptance of the ‘new me’; and (d) improving relationships.

#### 3.2.1. Mood

ADaPT was perceived to be important in helping to manage negative thoughts, which could lead to mood problems. Many participants acknowledged that managing their mood and wellbeing was a ‘work in progress’ and they had learned ways to avoid mood decline:

‘it’s not spiralling down [in negative thoughts]…I think that I have to do work on that.’ (P4, female).

The therapy was also perceived to help in lifting a participant’s mood through its promotion of participation in valued activities and connecting with others (e.g., talking to friends, writing to family, being present at family occasions, and attending the stroke/aphasia group). Others noted that the intervention assisted in alleviating anxiousness and stress by training strategies and techniques, such as ‘changing a view or outlook on a situation towards the positive side’ (P6, male); ‘building self-awareness of mood symptoms’ (P5, female); ‘using calm breathing’ (P4, female); and ‘keeping a thought record journal’ (P5, female).

A participant described a history of depression and being ‘more reclusive’ prior to the stroke and participation in ADaPT. A highly valued part of ADaPT was being supported by the therapist to build self-awareness of mood ‘warning signs’ and develop a depression relapse prevention plan:

‘Largely I think my mood has improved a lot [since completing the intervention]…a very positive impact, but I’m aware that I’m a bit up and down in my moods and so having a strategy in case something goes wrong…I love that.’ (P5, female)

#### 3.2.2. Communication

Participants reported that, while the ADaPT intervention had not improved or changed their communication functioning, they described ways they had come to accept communication changes due to aphasia. A common theme was that participants described having more confidence to communicate in different situations during and following CBT, such as talking over a meal with family, attending to stroke support group, or wishing a friend a happy birthday over the phone. They also described the impact of this on their social connection, mood, and wellbeing:

‘What a joy it was meeting the others from the stroke group…I’ve enjoyed that…I’ve been there three times so I feel I know them a bit better.’ (P5, female)

#### 3.2.3. Acceptance of the ‘New Me’

A common thread across the data was the participants’ perception that the therapist had helped them to accept changes in functioning post-stroke (communication, psychological, and physical abilities). In addition, they described ways that the therapist assisted participants to navigate new changes and identity with themselves and others, for example, through stroke and aphasia information provision:

‘Everybody else around me, you know, they don’t understand [stroke and aphasia]. [Therapist] she would understand those things because she knows what the problem is [aphasia]... she was actually explaining [what happened] in the brain as well…and then I was able to say to other people “No look this is the thing, and this is what happened.’ (P4, female)

A participant described how the therapist offered her a way to think and re-name herself post-stroke—by name and as ‘the new [participant’s first name]’ (P5, female). This participant reported this as one of the very positive things: an acceptance of her new identity. She was particularly appreciative of when the therapist took the time to speak with her and her husband about the grief following stroke and aphasia, and the reality of not returning to previous levels of functioning in terms of communication:

‘I think what was particularly helpful was addressing ongoing grief…I’m not the same as I was.’ (P5, female)

#### 3.2.4. Improving relationships

The majority of the participants reported the positive impact the ADaPT intervention had on managing relationships and social interactions. Three participants described the openness of the therapist to assist them in developing strategies (e.g., modifying thoughts, behaviour change) to manage pre-existing difficult relationships such as interactions with partners/spouses:

“I had to look at it differently [communication breakdowns with partner], different ways were written down [by therapist], which I brought home to try and go through that particular way of looking at it…I used to take myself out of the bad situation and look at other ways to talk to her.” (P6, male)

Participants also reported feeling more socially connected to others, less isolated and more confident as the therapist supported them to increase social participation and activities (e.g., attending aphasia or stroke groups, meeting friends). A participant (P2, male) described usually feeling ‘isolated’ but reported the therapist assisted him to join in more with meeting a ‘few people’ with his wife, an activity he would usually avoid prior to commencing the intervention.

Participants valued the therapist checking on their progress in social situations in between therapy sessions. They were able to share and express their feelings on how interactions occurred, feelings varied from those of frustration to satisfaction to feelings of pride, for example, initiating a phone call to a friend or joining the local library.

## 4. Discussion

Understanding the experiences and needs of stroke survivors with aphasia when participating in psychotherapeutic interventions is critical to establishing feasibility, acceptability, and identifying opportunities for improvement. To date, no other studies have explored the feasibility or perceived acceptability and experiences of people with aphasia participating in CBT facilitated by a psychologist and adapted for communication disabilities. Therefore, this current study focused on the experiences and needs of people with aphasia who were enrolled in the ADaPT single-case series study (60% of total sample) [18]. Overall, this sample strongly endorsed the intervention as acceptable and a positive experience. The modified CBT intervention was an acceptable therapy approach to all participants, including the participants with moderate to severe aphasia. All participants also considered appropriate and adequate communication support systems were provided. The intervention was tailored to participants’ goals and needs. Some participants preferred focusing on doing enjoyable activities, others on challenging their thinking patterns or in combination. Levels of independence in home-practice tasks varied: two participants were independent while the remaining participants required support of the therapist or spouse/carer. Five key areas were identified as helpful elements of therapy: doing enjoyable tasks; new ways of thinking; problem solving; working with the experienced therapist; and using telehealth. The participants described key areas for perceived gains in progress regarding mood, communication, accepting their new identity, and improving relationships. There was variation in experiences within each key area of therapy and within the theme of ‘making progress’; however, all participants were highly satisfied with and valued the therapy indicating that ADaPT was acceptable to them.

Participant perceptions around the ‘helpful elements of therapy’ support concepts of ‘active ingredients’ or ‘mediators’ for change in mood symptoms or outcomes within the psychological intervention [33,34]. Participants valued identifying and doing enjoyable activities, which aligns with previous research that concluded that behavioral activation is feasible and may reduce depressive symptoms after stroke and aphasia [35,36]. Focusing on increasing goal-directed and meaningful activities may lead to improved participation and feelings of ‘happiness’, ‘joy’, and ‘more confidence’, as described by participants in the current study. People with severe cognitive and/or communication difficulties may be challenged with cognitive aspects of CBT, hence the behavioral elements may be more engaging for them [36]. Another key element, ‘new ways of thinking’, was often a new concept to participants and helpful in changing from negative to more positive thinking patterns or accepting more realistic expectations of themselves post-stroke. This matches the previous research, which reported on two case studies (one with dysarthria and one with aphasia after stroke). Both participants had a reduction in anxiety symptoms immediately post-therapy and at follow up (3 months) [37]. These findings challenge the assumption that those with aphasia cannot engage with cognitive elements of CBT. A notable finding of this study is that participants with moderate to severe communication impairments (expressive and/or receptive skills) also felt ADaPT was a valuable therapy for them to participate in. This complements the preliminary evidence in the broader ADaPT study [18] that psychological therapy, such as CBT, though complex from some perspectives, can be modified to be accessible to people with aphasia, even those with moderate to severe impairments.

The variety of experiences and preferences within ADaPT also support the need for individually tailored interventions based on the person’s communication and psychological care needs, in addition to their therapy goals. This is consistent with the need for clinicians to ensure that people with aphasia are included in therapy goal setting and provided with communication support to make choices, negotiate, and agree upon goals and therapy activities [38]. This finding is consistent with solution-focused brief therapy, which views participants as the experts in their lives and enables them to achieve their goals and outcomes by drawing on their strengths, skills, and resources [39]. Furthermore, in the current study, the working relationship with the experienced therapist was highly valued and important to participants. Previous research confirms that speech pathologists report that verbal and non-verbal communication support are a vital factor to fostering a therapeutic alliance [40]. Of note, mental health practitioners report insufficient knowledge and training concerning aphasia, with a need for collaboration and interdisciplinary practice with speech-language pathologists [41]. It will be important for future research to understand the training, support, and supervision needs of ADaPT therapists to deliver therapy using modifications and approaches to support communication.

The changes participants perceived in accepting a new identity due to a disruption post-stroke are consistent with previous aphasia and identity research [4]. The findings of exploring identity as a helpful part of ADaPT aligns with the results from a subset of participants who experienced a renegotiation of post-stroke identity following solution-focused brief therapy [39]. This was possible through increasing self-respect, noticing personal strengths, and enabling the person to connect with their sense of who they are [39]. The experiences of ‘self under threat’ or discrepancy in identity following acquired brain injury are also central to the ‘Y-shaped’ model of rehabilitation [42]. The theories underpinning the model support the use of multiple approaches to address identity discrepancy, including a therapeutic focus on building social relationships and a self-awareness of thoughts, feelings, and abilities [42].

An additional notable finding of the current study is the perceived progress, in terms of communication confidence, improved mood, and knowledge of how to manage mood problems in the future (e.g., depression relapse prevention plan). These steps in progress enhanced the person’s ability to move forward, in-between and beyond therapy sessions in various ways, for example, involving themselves more in social interactions, which in turn assisted in reducing feelings of isolation. With the support of the therapist, most were able to build a level of self-awareness of their capabilities to make changes and intentionally seek ways to enhance their mood and wellbeing. This in-depth exploration of self-perceived progress and beneficial changes in mood should complement our understanding of CBT, which may be a helpful therapy post-stroke, despite the inconclusive nature of RCTs and meta-analysis in stroke research [14,43].

### 4.1. Clinical Implications

This study reports that people with aphasia highly value and accept ADaPT delivered by an experienced therapist. While a modification of the therapy may have been key, it is important to note that participants described the therapist’s understanding of aphasia and post-stroke mood problems as being important as well. In addition, experience that resulted in the therapist knowing how to personalize therapy in a flexible way to address current goals and everyday life problems was identified as critical. This indicates that simply providing the manual to therapists may not be enough to support the outcomes. Training would need to cover aphasia and stroke for those without this background, alongside the development of skills in supportive communication. The ADaPT therapist, a clinical neuropsychologist, has extensive clinical experience and skills working with people with neurological conditions, including stroke and aphasia. Professional clinical supervision by the experienced therapist will be a critical element to ensure the clinical integrity of ADaPT in future studies.

Participant suggestions for improvement in future included: enabling participants to have a clear understanding of the purpose of CBT to manage any potential expectations of language-based therapy; simplifying some handouts around concepts of grief and loss; and considerations to enhance connection and participation on telehealth.

### 4.2. Strengths, Limitations, and Future Directions

The study provides preliminary support for the broad applicability of ADaPT for people with aphasia; however, a future study with a larger sample of participants is required to confirm this. The ADaPT therapy was conducted during COVID-19, and so telehealth was used, which was also acceptable to participants as a mode of delivery. Despite the small sample size, there was a variation in time post-stroke onset and types/severities of aphasia of those participating in the ADaPT study, confirmed by comprehensive aphasia testing. The interviewer was independent of the therapy and single-case series study, with a background in speech pathology and communication support and access to people with aphasia. Thus, the interviewer was able to support communication to facilitate a rich and in-depth account of each participant’s experience. Using a reflexive thematic analysis enabled all interdisciplinary co-researchers to contribute their expertise in working with people after stroke, aphasia, and other acquired brain injuries (all from psychology and speech pathology disciplines). A limitation of the study was not including in-depth perceptions of close others (e.g., spouse/carer) who supported the person with aphasia throughout ADaPT and the limited diversity within the sample. Those who were unable to be interviewed or declined (n = 4) may have provided different views on their experiences, which may have influenced the findings in a different way. There was also a possible recruitment bias in the broader ADaPT study [18]. This study suggests that CBT within the ADaPT study is an acceptable approach, potentially warranting further investigation in a larger trial design. Developing appropriate training in the modified therapy will be a challenge for a major trial. Future qualitative research could include experts in aphasia, including those with lived experiences in co-producing aspects of the research, for example, topic guides for interviews. It could also include prospective interviews (prior to therapy) to understand the participant’s expectations of and hopes for therapy, with a possible comparison of perspectives post-therapy.

## 5. Conclusions

Modified CBT can be adapted for people with aphasia, including via telehealth, and it is acceptable to them. Participants identified key areas that were helpful to them and noted making progress in their mood, wellbeing, and communication confidence. The most useful parts of therapy included performing more enjoyable activities, modifying thoughts to be more helpful, and working with an experienced therapist knowledgeable in aphasia and stroke.

## Figures and Tables

**Table 1:** Participant characteristics.

Variables	Participant					
	P1	P2	P3	P4	P5	P6
Age (y)	58	70	59	71	69	64
Sex	Male	Male	Male	Female	Female	Male
Education (y)	15	17	12	17	15	9
Type of stroke	L ICA dissection	L MCA ischemic	L ischemic	Subdural hematoma	L hemorrhagic	L ICA occlusion
Tpo (y)	3	1	2	1	5	0.4
Aphasia quotient (WAB-R)	40.6 (severe)	78.2 (mild)	86 (mild)	71.1 (moderate)	89.48 (mild)	93.5 (mild)
Aphasia type	Wernicke’s	Anomic	Anomic	Conduction	Anomic	Anomic
Living with family?	Yes	Yes	No	Yes	Yes	Yes
Telehealth sessions (n)/10	10	10	9	10	10	9
Location	WA	NSW	Vic	NSW	NSW	Vic

Abbreviations: y = years, n = number, Tpo = time post-onset stroke, L = left, ICA= internal carotid artery, MCA = middle cerebral artery, WAB-R = Western Aphasia Battery-Revised total score and aphasia severity; WA = Western Australia, NSW = New South Wales, Vic = Victoria.

## Data Availability

This was a qualitative study exploring the experiences of participants when participating in therapy. Participant characteristic data, examples of data analysis, themes/subthemes derived from the data, and exemplar quotes from interview transcripts are provided in the manuscript and Supplementary Files.

## Supplementary Materials

The following supporting information can be downloaded at: https://www.mdpi.com/article/10.3390/healthcare12070771/s1, Table S1: Consolidated criteria for Reporting Qualitative studies (COREQ): 32-item checklist [25]; Table S2: interview topic guide; and Table S3: example of reflexive thematic data analysis process.

## References

[1] O’Halloran R., Worrall L., Hickson L. (2009). The number of patients with communication related impairments in acute hospital stroke units. Int. J. Speech Lang. Pathol..

[2] Baker C., Foster A.M., D’Souza S., Godecke E., Shiggins C., Lamborn E., Lanyon L., Kneebone I., Rose M.L. (2022). Management of communication disability in the first 90 days after stroke: A scoping review. Disabil. Rehabil..

[3] Flowers H.L., Skoretz S.A., Silver F.L., Rochon E., Fang J., Flamand-Roze C., Martino R. (2016). Poststroke Aphasia Frequency, Recovery, and Outcomes: A Systematic Review and Meta-Analysis. Arch. Phys. Med. Rehabil..

[4] Shadden B. (2005). Aphasia as identity theft: Theory and practice. Aphasiology.

[5] Northcott S., Moss B., Harrison K., Hilari K. (2016). A systematic review of the impact of stroke on social support and social networks: Associated factors and patterns of change. Clin. Rehabil..

[6] Kauhanen M.L., Korpelainen J.T., Hiltunen P., Määttä R., Mononen H., Brusin E., Sotaniemi K.A., Myllylä V.V. (2000). Aphasia, Depression, and Non-Verbal Cognitive Impairment in Ischaemic Stroke. Cerebrovasc. Dis..

[7] Morris R., Eccles A., Ryan B., Kneebone I. (2017). Prevalence of anxiety in people with aphasia after stroke. Aphasiology.

[8] Hilari K. (2011). The impact of stroke: Are people with aphasia different to those without?. Disabil. Rehabil..

[9] Zanella C., Laures-Gore J., Dotson V., Belagaje S. (2023). Incidence of post-stroke depression symptoms and potential risk factors in adults with aphasia in a comprehensive stroke center. Top. Stroke Rehabil..

[10] Baker C., Worrall L., Rose M.L., Hudson K., Ryan B., O’Byrne L. (2018). A systematic review of rehabilitation interventions to prevent and treat depression in post-stroke aphasia. Disabil. Rehabil..

[11] Ryan B.J., Clunne S.M., Baker C.J., Shiggins C., Rose M.L., Kneebone I.I. (2022). A systematic review of non-drug interventions to prevent and treat anxiety in people with aphasia after stroke. Disabil. Rehabil..

[12] Broomfield N.M., Laidlaw K., Hickabottom E., Murray M.F., Pendrey R., Whittick J.E., Gillespie D.C. (2011). Post-stroke depression: The case for augmented, individually tailored cognitive behavioural therapy. Clin. Psychol. Psychother..

[13] DeRubeis R.J., Hollon S.D., Amsterdam J.D., Shelton R.C., Young P.R., Salomon R.M., O’Reardon J.P., Lovett M.L., Gladis M.M., Brown L.L. (2005). Cognitive Therapy vs Medications in the Treatment of Moderate to Severe Depression. Arch. Gen. Psychiatry.

[14] Wang S.B., Wang Y.Y., Zhang Q.E., Wu S.L., Ng C.H., Ungvari G.S., Chen L., Wang C.X., Jia F.J., Xiang Y.T. (2018). Cognitive behavioral therapy for post-stroke depression: A meta-analysis. J. Affect. Disord..

[15] Ahrens J., Shao R., Blackport D., Macaluso S., Viana R., Teasell R., Mehta S. (2022). Cognitive-behavioral therapy for managing depressive and anxiety symptoms after stroke: A systematic review and meta-analysis. Top. Stroke Rehabil..

[16] Stroke Foundation (2020). National Stroke Audit Rehabilitation Services 2020.

[17] (2021). James Lind Alliance Priority Setting Partnership for Stroke.

[18] Tjokrowijoto P., Thomas S., Kneebone I., Ryan B., Stolwyk R.J. (2024). Aphasia, Depression, and Psychological Therapy (ADaPT): A single case design evaluation of a modified cognitive behavioural therapy to treat depressive symptoms in stroke survivors with aphasia. Neuropsychol. Rehabil..

[19] Sekhon M., Cartwright M., Francis J.J. (2017). Acceptability of healthcare interventions: An overview of reviews and development of a theoretical framework. BMC Health Serv. Res..

[20] Braun V., Clarke V. (2022). Thematic Analysis: A Practical Guide.

[21] Braun V., Clarke V. (2022). Conceptual and design thinking for thematic analysis. Qual. Psychol..

[22] Cheng B.B.Y., Ryan B.J., Copland D.A., Wallace S.J. (2022). Prognostication in Poststroke Aphasia: Perspectives of Significant Others of People With Aphasia on Receiving Information about Recovery. Am. J. Speech Lang. Pathol..

[23] Gibson B., Umeh K., Davies I., Newson L. (2021). The best possible self-intervention as a viable public health tool for the prevention of type 2 diabetes: A reflexive thematic analysis of public experience and engagement. Health Expect..

[24] Luck A., Rose M. (2007). Interviewing people with aphasia: Insights into method adjustments from a pilot study. Aphasiology.

[25] Tong A., Sainsbury P., Craig J. (2007). Consolidated criteria for reporting qualitative research (COREQ): A 32-item checklist for interviews and focus groups. Int. J. Qual. Health Care.

[26] Kertesz A., Raven J.C. (2007). The Western Aphasia Battery Revised, revised ed..

[27] Turner-Stokes L., Kalmus M., Hirani D., Clegg F. (2005). The Depression Intensity Scale Circles (DISCs): A first evaluation of a simple assessment tool for depression in the context of brain injury. J. Neurol. Neurosurg. Psychiatry.

[28] Ryan B., Kneebone I.I., Rose M.L., Togher L., Power E., Hoffman T., Khan A., Simmons-Mackie N., Carragher M., Worrall L. (2023). Preventing depression in aphasia: A cluster randomised control trial of the Aphasia Action Success Knowledge (ASK) program. Int. J. Stroke.

[29] Worrall L., Ryan B., Hudson K., Kneebone I.I., Simmons-Mackie N., Khan A., Hoffman T., Power E., Togher L., Rose M. (2016). Reducing the psychosocial impact of aphasia on mood and quality of life in people with aphasia and the impact of caregiving in family members through the Aphasia Action Success Knowledge (Aphasia ASK) program: Study protocol for a randomized controlled trial. Trials.

[30] Wilson C., Kim E. (2021). Qualitative data collection: Considerations for people with Aphasia. Aphasiology.

[31] Lyons R., McAllister L. (2019). Qualitative Research in Communication Disorders: An Introduction for Students and Clinicians.

[32] Shiggins C., Ryan B., O’Halloran R., Power E., Bernhardt J., Lindley R.I., McGurk G., Hankey G.J., Rose M.L. (2022). Towards the Consistent Inclusion of People With Aphasia in Stroke Research Irrespective of Discipline. Arch. Phys. Med. Rehabil..

[33] Lemmens L.H.J.M., Müller V.N.L.S., Arntz A., Huibers M.J.H. (2016). Mechanisms of change in psychotherapy for depression: An empirical update and evaluation of research aimed at identifying psychological mediators. Clin. Psychol. Rev..

[34] Lorenzo-Luaces L. (2023). Identifying active ingredients in cognitive-behavioral therapies: What if we didn’t?. Behav. Res. Ther..

[35] Thomas S.A., Walker M.F., Macniven J.A., Haworth H., Lincoln N.B. (2013). Communication and Low Mood (CALM): A randomized controlled trial of behavioural therapy for stroke patients with aphasia. Clin. Rehabil..

[36] Thomas S.A., Drummond A.E., Lincoln N.B., Palmer R.L., das Nair R., Latimer N.R., Hackney G.L., Mandefield L., Walters S.J., Hatton R.D. (2019). Behavioural activation therapy for post-stroke depression: The BEADS feasibility RCT. Health Technol. Assess..

[37] Kneebone I.I., Jeffries F.W. (2013). Treating anxiety after stroke using cognitive-behaviour therapy: Two cases. Neuropsychol. Rehabil..

[38] Worrall L., Sherratt S., Rogers P., Howe T., Hersh D., Ferguson A., Davidson B. (2011). What people with aphasia want: Their goals according to the ICF. Aphasiology.

[39] Northcott S., Simpson A., Thomas S., Barnard R., Burns K., Hirani S.P., Hilari K. (2021). “Now I Am Myself”: Exploring How People With Poststroke Aphasia Experienced Solution-Focused Brief Therapy Within the SOFIA Trial. Qual. Health Res..

[40] Lawton M., Sage K., Haddock G., Conroy P., Serrant L. (2018). Speech and language therapists’ perspectives of therapeutic alliance construction and maintenance in aphasia rehabilitation post-stroke. Int. J. Lang. Commun. Disord..

[41] Strong K.A., Randolph J. (2021). How Do You Do Talk Therapy With Someone Who Can’t Talk? Perspectives From Mental Health Providers on Delivering Services to Individuals With Aphasia. Am. J. Speech-Lang. Pathol..

[42] Gracey F., Evans J.J., Malley D. (2009). Capturing process and outcome in complex rehabilitation interventions: A “Y-shaped” model. Neuropsychol. Rehabil..

[43] Lincoln N.B., Flannaghan T. (2003). Cognitive behavioral psychotherapy for depression following stroke: A randomized controlled trial. Stroke.

