# Supplementary material for: Aphasia Depression and Psychological Therapy (ADaPT): Perspectives of People with Post-Stroke Aphasia on Participating in a Modified Cognitive Behavioral Therapy
Source: Healthcare (Basel). 2024 Apr 2;12(7):771. doi: 10.3390/healthcare12070771 (PMC11012076; doi:10.3390/healthcare12070771)
Supplement: Supplementary file 1 [file healthcare-12-00771-s001.zip › healthcare-2917305-supplementary.pdf]

Supplementary file S1 Table S1 COnsolidated criteria for REporting Qualitative studies (COREQ): 32-item checklist [25]

No	Item	Guide questions/description
**Domain 1: Research team and reflexivity**		
Personal Characteristics		
**1.**	Interviewer/facilitator	C.Baker
**2.**	Credentials	Speech Pathologist, PhD
**3.**	Occupation	Speeech Pathology Research and Clinical Practice Lead
**4.**	Gender	Female
**5.**	Experience and training	Extensive combined experiences in clinical, research and teaching roles over 22 years.<br>Research team composed of psychologists and speech pathologists
Relationship with participants		
**6.**	Relationship established	None
**7.**	Participant knowledge of the interviewer	Speech Pathologist and co-researcher on ADaPT study
**8.**	Interviewer characteristics	Speech pathology background with research focus area of psychological care after aphasia
**Domain 2: study design**		
Theoretical framework		
**9.**	Methodological orientation and Theory	Reflexive thematic analysis [18,19]
Participant selection		
**10.**	Sampling	Purposive sampling with maximum variation for sex, age, aphasia severity
**11.**	Method of approach	Invitation via email / phone / videoconference
**12.**	Sample size	Six
**13.**	Non-participation	Seven were invited to participate. One participant was considered to decline as they failed to respond to 3 invitations to do so
Setting		
**14.**	Setting of data collection	Participants' homes (all via videoconference); one was interviewed in person at the clinic
**15.**	Presence of non-participants	Yes, two participants had their spouse present to assist as a communication support partner
**16.**	Description of sample	See Table 1.

Data collection		
**17.**	Interview guide	The topic guide included open ended questions and prompts. It was reviewed by all co-authors
**18.**	Repeat interviews	None
**19.**	Audio/visual recording	All interviews were video recorded then transcribed verbatim
**20.**	Field notes	Field notes were taken by the primary researcher after the interviews
**21.**	Duration	Average duration was 56 minutes
**22.**	Data saturation	Data saturation was not relevant to reflexive thematic analysis. Not complete data saturation.
**23.**	Transcripts returned	Transcripts were not returned
**Domain 3: analysis and findings**		
Data analysis		
**24.**	Number of data coders	There was one primary coder (CB) and a peer-review process of all codes with the research team
**25.**	Description of the coding tree	See Supplemental file Table S3.
**26.**	Derivation of themes	Themes were derived from the data
**27.**	Software	Microsoft Excel
**28.**	Participant checking	Participant checking was not conducted, rather participants provided confirmation of messages / meaning throughout and at the end of the interview
Reporting		
**29.**	Quotations presented	Quotations from different participants were presented
**30.**	Data and findings consistent	Data and findings consistent
**31.**	Clarity of major themes	Two major / core themes clearly identified ‘Helpful elements of therapy’ and ‘Making progress’
**32.**	Clarity of minor themes	Minor themes identified within each core theme

Supplementary file S2 Table S2: Interview topic guide

Qualitative study Following completion of the modified cognitive behaviour therapy (ADaPT), the participant will be invited to complete a semi-structured interview which will explore their experience and perspectives of the therapy. Supported communication strategies will be used throughout the interview (using pen and paper to write/draw; gestures; objects; yes/no questions).

Acknowledgement This interview guide is a modified version of the Action Success Knowledge (ASK) clinical trial qualitative process evaluation that was written by Professor Emma Power, University of Technology Sydney (Ryan et al., 2023; Worrall et al.,2016).

Interview guide

A. Opening Introduction

CONTEXT
Today I would like to hear about your experiences in the CBT program. I was not part of providing the CBT. You can provide open and honest feedback about your experiences. There are no right or wrong answers. We are interested in your experience and views.
It's important that we understand what it was like for you to be involved in the study.
The information you provide will help us to understand your experience and improve the program. I will videorecord this discussion – is this ok?

B. Reach/motivation to be involved in the trial

CONTEXT	QUESTION	MATERIALS
The first questions are about why you got involved in the CBT program study.		Pen, paper, key words, drawings, screen share as needed.
Think back to the very beginning, before the therapy started.		
You did not have to do this study, it was your choice and you volunteered.	• Why did you decide to participate in the study?	
	• What motivated you to get involved?	
	• What did you expect from the therapy?	

C. Orientation to the therapy

CONTEXT	MATERIALS
Now, I would like you to tell me about the CBT program you were part of	
We are interested in your participation in CBT not other therapy or rehabilitation experiences in other settings	
You completed the sessions at the clinic / via telepractice using Zoom with [therapist name].	SHOW VISUAL TIMELINE 10 sessions (in person/telehealth etc using Zoom)

D. Confirm participation

CONTEXT	QUESTION	MATERIALS
In the sessions, you worked on different things	Tell me about what you did in CBT…<br><br>Prompt if needed:<br>Goal setting?<br>Type of therapy tasks?<br>Monitor of changes / progress?	Participant can show their own therapy materials if this helps with communication
	How did you feel about participating in CBT….<br><br>Prompt if needed:<br>Level of effort / difficulty / demand?	

E. Fidelity: dose received

CONTEXT	QUESTION	MATERIALS
Let’s now think about the different parts of the CBT that you received	• Overall, how do you feel about the amount of CBT you received?	Use 0-10 scale<br>0=not enough<br><br>10=too much
You received 10 CBT sessions	• What did you think about the number of sessions you had?	0-10 scale
	• How did you feel about the length of the sessions? (longer/shorter/just right)	Use 0-10 scale

	• Did you miss any sessions?	
	• What led to you missing these sessions?	
[therapist name], the therapist may have given you work to do at home.	• Tell me about any home practice / between session tasks…<br>• Did you continue with work at home?<br>• Was home practice useful / helpful or not?<br>• What was good / less good about home practice?	

F. Implementation: barriers/facilitators

CONTEXT	QUESTION	MATERIALS
We want to hear what helped or didn't help during the CBT program.		
	• Tell me what helped you to participate in CBT…<br>• What made it easier to participate?	Use green tick diagram to convey helpful / facilitators
You kept going with the CBT to the end of the program.	• Did anything or anyone help you to keep going?	
	• Tell me what didn't help you to participate in CBT…<br>• What made it difficult to participate?	Use red cross diagram to convey barriers
If we were to offer the treatment in the future	• Would you participate again?	
	• What could make it easier to participate in CBT?	
	• Would you recommend this CBT program to other people with stroke and aphasia?	

G. Participant response: perceived changes and unexpected consequences

CONTEXT	QUESTION	MATERIALS
We are interested in hearing about what impact CBT might		

have had on your life and the way you live.		
	• Did the intervention have a positive or negative impact on you? Tell me about this…	
	• Did you find the intervention helpful/ unhelpful?	
	o Can you give me an example/s?	
	• Have you noticed any changes in the way you feel in your mood after being involved in CBT?<br>• Have you noticed any changes in the way you feel about your life after being involved in CBT? (e.g., The way you think about yourself, your relationships, things that you do…?)	
	• Was there anything in particular from the treatment that influenced the way you have been feeling?	
	o If there were benefits did these continue after the study or have they stopped? Why?	
Overall when you think about the CBT program	• What do you think are the two MOST useful and two LEAST useful parts of the program?	
	• Did anything surprise you in the program?	
	o Was there anything unexpected? Anything less good / negative?	
	• Is there anything else you think we should include in the therapy? Any changes to materials?	
	• How was [therapist name] at working with you?	Show visual scale of 1-5<br>1=poor<br>2= fair<br>3=good<br>4=very good<br>5=excellent

H. Context of COVID-19 and precautions

CONTEXT	QUESTION	MATERIALS
We want to understand your experience of participating in CBT during COVID-19	How did you find participating in CBT during COVID-19?	
	• Were there good/less good things about therapy during COVID?	
	o	
	• Were there any impacts of COVID-19 associated with your mood? If so, please explain<br>• Were there any impacts of COVID-19 associated with participating in CBT? If so please explain<br>Consider positive and negative impacts	

I. CBT via telehealth using Zoom videoconference

CONTEXT	QUESTION	MATERIALS
We offered CBT via telehealth using Zoom due to COVID-19 precautions.<br>We want to understand your experience of using Zoom for CBT.	• Tell me about your experience of using Zoom…<br>• What was good/less good?<br>• What was difficult?<br>• How was your working relationship with [therapist name] via Zoom?<br>• What are TWO most useful things about Zoom.<br>• What are TWO least useful things about Zoom.<br>• Would you recommend using Zoom again for CBT?	

J. Confirmation and summary

CONTEXT	QUESTION	MATERIALS
Interviewer will summarise main points or standout issues discussed during the interview, to check interpretation	• Let me summarise what I have understood from our conversation<br>• Did I get it right?<br>• Is there anything else you would like to add?	Paper and pen to present main points discussed

Ryan, B., Kneebone, I. I., Rose, M. L., Togher, L., Power, E., Hoffman, T., Khan, A., Simmons-Mackie, N., Carragher, M., & Worrall, L. (2023). Preventing depression in aphasia: A cluster randomised control trial of the Aphasia Action Success Knowledge (ASK) program. International Journal of Stroke, 18(8) 996-1004. https://doi.org/10.1177/17474930231176718

Worrall, L., Ryan, B., Hudson, K., Kneebone, I. I., Simmons-Mackie, N., Khan, A., Hoffman, T., Power, E., Togher, L., & Rose, M. (2016). Reducing the psychosocial impact of aphasia on mood and quality of life in people with aphasia and the impact of caregiving in family members through the Aphasia Action Success Knowledge (Aphasia ASK) program: Study protocol for a randomized controlled trial. Trials, 17, 153 https://doi.org/10.1186/s13063-016-1257-9

Supplementary file S3 Table S3: Example reflexive thematic data analysis process

Participant Quote	Initial Codes	Codes	Sub-theme	Core Theme
"the things that gave me joy…like listening to music…"	• Joy from activities	• Doing valued activities lifts mood	• Doing enjoyable things	• Helpful elements of therapy
"I'll come across a couple of magpies…I'll talk to her…feed her…that makes me happy"	• Happy when with birds, dogs			
"I am thinking about the good side, the positive side…rather than, I just thinking of the worst"	• Changing from thinking of the worst	• Changing unhelpful thoughts	• New ways of thinking	
"... I remember a day that I met up with some old friends from work unexpectedly, and I wasn't prepared to in any way... [therapist helped with] modifying my thoughts and my negative thoughts and making more realistic, accepting more my... and pointing out the things that I did well"	• Changing from negative to realistic thinking			
